# A model to foster and facilitate trust and trusting relationships in the nursing education context

**DOI:** 10.4102/hsag.v26i0.1645

**Published:** 2021-12-03

**Authors:** Ellie C. van Dyk, Gisela H. van Rensburg, Elsie S. Janse van Rensburg

**Affiliations:** 1 Department of Health Studies, Faculty of Human Sciences, University of South Africa, Pretoria, South Africa

**Keywords:** conducive teaching, grounded theory, learning, model, nursing education context, trust

## Abstract

**Background:**

In the nursing education context, the fostering of trust and establishing trusting relationships are important facets of teaching and learning.

**Aim:**

The purpose of the study was to understand trust and trusting relationships in teaching and learning, and to develop a model to foster and facilitate trust and trusting relationships in the nursing education context.

**Setting:**

Two nursing education institutions were purposively sampled that offered a comprehensive programme in nursing.

**Methods:**

A grounded theory study was conducted with the aim to develop a model for trust and trusting relationships in teaching and learning. Purposive, convenience and theoretical sampling of participants were applied. The sample size consisted of 5 educators and 23 students at a university and 9 educators and 37 students at a public nursing education institution. Charmaz’s methods were used for the data analysis. Collection and analysis of data were conducted concurrently.

**Results:**

Data of views from the educators and students on trust were synthesised. Themes identified were: professional relations, expectations of the role players in nursing education, creating a conducive teaching and learning context, and outcomes of lack of trust. From the findings, a substantive model to foster and facilitate trust and trusting relationships in nursing education was developed.

**Conclusion:**

The study contributed to understanding trust in nursing education. Recommendations focus on fostering and facilitating self-trust and trusting relationships and also on how the model could be implemented in nursing education.

**Contribution:**

Fostering and facilitating trust and trusting relationships in the nursing education context will enhance a trusting culture and contribute to the quality of teaching and learning.

## Introduction

Trust and trusting relationships are the cornerstone of human relationships and are of great importance in the lives of humans. Erikson (1959) as cited in Van Vuren (2012) indicated that the formation of basic trust happens during the first psychosocial developmental phase (Kalat 2016). Interpersonal relations develop based on trust that forms the foundation of human relationship over the lifespan of a person. Many researchers consider trust as an important topic (Bencsik & Machova 2016) especially in organisations where collaboration and engagement are essential to achieve organisational success (Covey & Link 2012). Globally in academic disciplines, trust is considered a crucial matter (Uslaner 2016) and it is critical to have positive trusting cultures in educational environments (Erdogan 2016). Societies’ expectations about trust in schools are changing as trust affects education and the atmosphere of educational institutions (Tschannen-Moran 2014). Trust in teaching and learning are not only devoted to relationships but extend to the educational environment, characteristics of educators and students such as behaviours, standards, attitudes, beliefs and expectations (Van Maele, Forsyth & Van Houtte 2014).

The fundamental task in a nursing education environment is the education and training of nurses to become competent professional nurses. There is daily interaction amongst educators, students and professional nurses creating learning opportunities to transfer knowledge and skills. A lack of trust may negatively influence educators’ and students’ self-worth and academic accomplishments. During an inquiry into triggers of high failure rate amongst nursing students in one of the provinces of South Africa, trust issues were highlighted as a trigger (Department of Health [s.a]). Students indicated triggers such as lecturers’ unapproachability, negative attitudes, impatience and poor academic support. Inappropriate clinical support and accompaniment were issues that hampered sufficient clinical learning. Favouritism of certain students, and unequal treatment of students, hampered fairness and good interpersonal relationships (Department of Health [s.a]). As a result of poor examination marks, students requested to view the marked examination scripts. They were only satisfied with the marking process and their examination result after reviewing the script and not finding any marking errors. In turn, educators felt that their standards were questioned but concerns about inconsistency amongst educators may cause a barrier in the trusting relationships and trust in the standards of the programme. Academic support to students was not prioritised (Department of Health [s.a]). Concerns were raised about nursing values and the standard of patient care for some students who completed the programme.

Educators revealed that professional nurses neglected their teaching responsibilities when students are placed for clinical learning opportunities (Van Dyk, Van Rensburg & Janse van Rensburg 2016). The supervision negligence of professional nurses during clinical placements put additional demands on educators and influences the quality of student who completed the nursing programmes. In addition, poor support to students from professional nurses in the clinical services resulted in a lack of appropriate role modelling as expressed by participants. Nursing is a profession with trustworthiness and mistrust in the education and training of students nurses will hamper the professional image of nursing education. The production of competent professional nurses require a high standard of education and training.

Against this backdrop, trust in the nursing education context is crucial for teaching and learning. The research problem reflected that poor academic performances of student nurses may be because of a lack of trust and trusting relationships in nursing education. Unmotivated students with poor academic performance pose a risk to the nursing standards and image of the nursing profession. The research questions informing this study were: ‘How do trust and trusting relationships affect teaching and learning in nursing education?’ and ‘Under which conditions do trust and trusting relationships develop in the theoretical and clinical environment?’

The aim of this study was to explore and understand trust and trusting relationship in nursing education to develop a substantive model to foster and facilitate trust and trusting relationships in the nursing education context. The following objectives guided the study: explore and describe educators’ and students’ views on trust and trusting relationships in the nursing education context, develop a model to foster and facilitate trust and trusting relationships in the nursing education context and make recommendations to implement the model that will enhance trust in the nursing education context. This article aims to describe the model for trust in education and learning that was developed from the findings of the study, and how it could be applied to higher education institutions that offer training of healthcare professionals. At the core of the model is self-trust and relation trust. Components of the model create an alert to professional conduct and an awareness of striving to create a theoretical and clinical environment conducive to enhance teaching and learning.

## Research design and method

### Research design

A qualitative approach and grounded theory design were used to explore the views of educators and students in order to develop the model. Qualitative research describes meanings, experiences and perspectives of individuals with a systematic and subjective approach (Creswell & Cresswell 2018). Knowledge development in grounded theory has a subjective approach as a result of the involvement in the idiosyncratic world of participants (Charmaz 2014). The model was developed from the findings, literature review and a process of inductive and deductive reasoning based on the concept synthesis steps of Walker and Avant (1995). The model was built on processes of co-construction of meaning during the data collection and analysis processes as described by Charmaz (2014). Given the nature of grounded theory research, the researcher reviewed and applied relevant literature simultaneously during the data collection and analysis. Links were drawn between concepts during three phases of coding (initial, focused and theoretical). Walker and Avant (1995) and Charmaz (2014) described the development of a substantive theory or model as processes that demonstrate and relate the properties of final concepts. The final processes of inductive reasoning and concept synthesis provided a holistic graphic presentation of the model.

### Research method

The research method refers to the population, data collection, analysis and measures of trustworthiness and ethical principles.

#### Research setting

The research was conducted in one of the provinces of South Africa at a university nursing education institution (NEI) and a public multi-campus NEI with three campuses. The three campuses of the public NEI were in urban, semi-rural and rural districts. The 4-year nursing programme (R425) as stipulated by the South African Nursing Council (SANC) was offered at all of the NEIs.

#### Population

The population refers to all the individuals who meet the inclusion and eligibility criteria (Brink, Van der Walt & Van Rensburg 2018) and those persons who are reasonably accessible by the researcher is known as the target population (Gray, Grove & Sutherland 2016). Educators and students were the target population. The criteria for the educators were: employed educators at the specified two accredited NEIs with an additional qualification in nursing education, educators with theoretical teaching and clinical accompaniment experience, educators who are clinically experienced and up-to-date with the clinical settings. Students’ eligibility criteria were nursing students in their third or fourth year of study of the 4-year nursing programme at the specified institutions. The motivation for including those students was that they were exposed to different teaching and learning environments during their training.

Samples in grounded theory studies range between 20 and 30 people because of theoretical sampling (Polit & Beck 2016). Purposive and theoretical sampling were applied. Participating educators were purposively selected based on the eligibility criteria, their willingness and availability to participate. Students were selected using a convenience sampling technique and considered based on the inclusion criteria. In grounded theory, the researcher applies theoretical sampling when specific data are needed to refine categories and an emerging theory is developed (Charmaz 2014). The researcher utilised theoretical sampling, focused on the concepts and emerging codes until data saturation was evident and no new properties emerged.

#### Data collection

Data collection commenced after ethical clearance was obtained and written informed consent from the participants for participation and audio–voice recordings of interviews. Data collection commenced from September to November 2013 at public nursing education institutions and from February to April 2014 at the University NEI. The findings of this study are still relevant given the literature and results of recent studies. These studies highlight the relevance and applicability of the data and relational statements that are portrayed in the model. Trust and trusting relationships are part of interaction amongst people. Knowles (2021) stated ‘Trust is the foundational principle that holds all relationships together’. Developing trusting relationships is essential during education and training. Trust is developed through interaction between educators and students. When trusting relationships are evident, students will follow the educator. Cognitive and affective components are attached to trust in the nursing education environment (Liu 2020). Barrett and Harris (2019) identified three core themes, namely ‘giving oneself’, ‘being competent’ and ‘having integrity’ that reinforce the development of trust in nursing education. Linked with these themes, Froneman, Du Plessis and Koen (2016) mentioned that loving and caring, mutual respect, openness and willingness to support, improve the educator–student relationship. Educators’ characteristics and competencies emphasise specific academic characteristic and credible clinical skills. The World Health Organisation (2016) maintained that the nurse educator should have the ability to foster a relationship of mutual trust and respect, and demonstrate interest and mutual respect for students. Moral values (Froneman et al. 2016), adherence to professional codes of ethical practice (WHO 2016), goodwill actions, expected characters and virtues are cornerstones that are valued for the development of trust and trusting relationships (Dinc & Gastmans 2012). These components mentioned by the researchers are contained in the model that was developed for this study.

Data were collected through unstructured interviews and focus group interviews. In-depth face-to-face interviews are unstructured (Lichtman 2014) and focus group interviews aim to promote self-disclosure of feelings on a particular topic in a shorter period (Brink et al. 2018). Five educators from the university and nine educators from the public nursing education institution participated in unstructured in-depth face-to-face interviews. A total of 60 students, 23 from the university and 37 from the public NEI participated in 14 focus group interviews. Data saturation was reached in the face-to-face interviews and the focus group interviews. Field notes were made before, during and after the face-to-face and focus group interviews. Data collection and analysis were employed simultaneously. The length of the interviews varied between 50 and 60 min. Participants gave written informed consent for participation and the voice-recordings of interviews.

#### Data analysis

Charmaz’s (2014) three stages of data analysis: initial, focused and theoretical coding were used. This study was interpretive in nature and was guided by symbolic interactionism and constructivism with an inductive approach that started with the first individual interview and focus group interview. The researcher used constant comparison to reduce data and develop categories and codes (Johnson & Christensen 2012). The two data sets from educators and students were constantly compared and relevant literature were integrated to develop the model.

The researcher developed the model using theoretical coding and the concept synthesis steps of Walker and Avant (1995). Reduction of data and an iterative, cyclic process were applied, meaning that saturation was achieved by going forward and backward until the properties of final concepts were identified. Comparison of concepts and the integration of literature ensured triangulation and verification processes. The latter were constantly performed until no new concepts emerged. The clustering of concepts and synthesis thereof, sub-categories and categories became clear. From the synthesis, five themes that are related to each other emerged. These themes represent the components of the model that were combined for the final model for trust in an education and learning environment.

Based on the data obtained from participants, field notes and literature, a substantive model was developed. Authorities in the fields of general and nursing education, graphic design and psychology evaluated the model for its clarity, consistency and simplicity; relevance and appropriateness; comprehensiveness; generalisability and adaptability; usefulness and practicality; accessibility; importance for education, transferability; practice and research. Reviewers were provided with a document explaining the model and the schematic presentation of the model. The review document was in the form of a Likert scale (acceptable as is, acceptable with changes, not acceptable or needs major revision). They were also provided with an option to make comments. Based on the feedback of the reviewers, the model was refined and finalised.

## Measures of trustworthiness

The trustworthiness model of Lincoln and Guba’s (1985) as cited in Holloway and Weeler (2010) was utilised in this study (Korstjens & Moser 2018). Credibility such as the truth of the participants’ views, which was supported through the first author’s engagement during interviews and observations and tracking of audit trails was evident. Through memoing, an inquiry audit that kept track of the development of the model, dense descriptions of data from the in-depth interviews and focus group interviews until data saturation of categories and sub-categories, transferability was obtained. Dependability is supported by the transferability of findings, namely if a study will be conducted in similar conditions, similar data will be obtained. Conclusions and interpretations made from the dialogues of participants and in-depth data confirm the criterion of confirmability. Authenticity was attained through the participants’ views, feelings and precise verbatim quotes relating to trust and trusting relationship.

### Ethical considerations

To prevent any exposure and discomfort of participants, the basic ethical principles of research were followed. Brink et al. (2018) emphasised the implementation of the ethical principles of beneficence, autonomy and justice. Ethical issues in this study including informed confidentiality, consent, prevention of harm and anonymity were considered and applied after ethical clearance were obtained from the higher educational institutions (Ref HSHDC/114/2012; ECUFS Nr 167/2013). Permission was attained from the relevant stakeholders, namely the Department of Health, the participating university and the principal and heads of campuses of the public nursing college to conduct the study. Autonomy was assured by obtaining informed consent from participants. Justice was applied by ensuring a fair selection of participants according to an eligibility criteria. Beneficence was ensured by voluntary participation and explaining the benefits of the study and ensuring that the participants knew their rights linked to participation and withdrawal from the study.

## Results

The themes that emerged were: professional relations, expectations of the role players in nursing education, creating a conducive teaching and learning environment and outcomes of trust or lack of trust. Table 1 reflects verbatim quotes from participants on themes used to develop a model to foster and facilitate trust and trusting relationships in the nursing education environment.

**TABLE 1:** Verbatim quotes in categories and sub-categories.

Categories	Responses
**Professional relations**	
Self-trust	‘Number one: you must have trust in yourself, because if you are having self-trust you appear trustful to other people … that is a need in front of the students.’ (Participant 3, female, nurse educator)
	‘And this will also improve the self-trust and the trust of their patients. If you trust yourself and you can improvise such things, then you will present your patient with trust.’ (Participant 1, female, nurse educator)
Relation trust	‘Communication has a very important role in trust because the more you communicate with someone it is then that you know the person, you get to understand the way they are doing things. When you are seeing someone is doing something and you do not communicate to that person and understand why they are really doing that, you won’t trust the person, because of the way they are doing some things … and it improves the interpersonal relationship.’ (Participant 8, female, student nurse)
	‘If there is a trust relationship … then your students excel. It is just that they then have confidence in themselves and they know that you trust them and they grow both academically and as a person.’ (Participant 5, female, nurse educator)
**Expectations of the role players in nursing educ ation**	
Professional credibility	‘Look, your professionalism is the foundation of everything. If it is not there, there is nothing. Without professionalism there cannot be trust between anybody or group. Because professionalism says that you are a person with certain values, certain ethical norms, certain discipline. This behaviour can be expected of you.’ (Participant 3, female, nurse educator)
	‘If there is no professionalism then I do not trust the rest. If a person throws away the basic ethical things, then for all I know that person is no longer competent … Once you have that image, it gives you trust for the rest. If you have that basic professionalism, you have the skill and you will be professional enough to acquire those competencies.’ (Participant 11, female, nurse educator)
Competencies	‘… to give them information, knowledge up to date, recent practice, that is expected of them; useful, that they can use it in the practical areas and do not learn things that they cannot use.’ (Participant 3, female, nurse educator)
	‘She must also be an expert in the area where she is working, because if the students know more than the sister, the trust will be broken.’ Participant 1, female, nurse educator
Professional virtues	‘The issue that affects the trusting relationship between the lecturer and students is etiquette, the most important aspect, because the way I see it is like we are no more emphasising nursing etiquette … Etiquette is actually guiding the students towards a good lecturer and student relationship and that also guides the student towards a good relationship.’ (Participant 3, female, nurse educator)
	‘Your values of your profession will cause you to adhere to them or not. There must be a marriage between the values of the profession and your values and I must recognise that this is so and you must be able to see it in me, then we can have a trust relationship. More so, if I do not see the professional values, then I cannot trust you.’ (Participant 14, female, nurse educator)
Congruency	‘I think we need, we need to have discipline and we need to have consistency. Because, if you tell somebody that you want something and they do not do it, and they get away with it, then you need discipline and you need consistency.’ (Participant 3, female, nurse educator)
	‘So everything must come from this professional behaviour. You cannot just do what you want to, one day this and the next that, you have guidelines. I believe a professional person is someone who can think quickly, has knowledge, knows the policies and procedures, knows how things are performed, because that is the only way in which she can do things correctly in a professional capacity. It also lies within yourself, your self-respect, your ethical environment and what is right is right, fairness … you know such professional ways of doing things.’ (Participant 4, female, nurse educator)
**Creating a conducive teaching and learning environment**	
Theoretical environment	‘… you must be very creative how you present this subject to keep the student interested in the content, and use different techniques … method teaching … Use all the methods like, using lecture method, group discussion and presentations. We are using debates, depending on the content that needs to be understood and absorbed by the student. We are using … sometimes we go to the extent using quizzes.’ (Participant 3, female, nurse educator)
	‘You know the trust from our students is gone, with our presentations, because we are still using the old style, we do not have resources. The classroom environment itself, you can go to the classroom … I mean, you go and see it is just a hall. It is a class, an old table standing there and nothing else. Then you have to use either the books or present from whatever you are presenting. We do not have resources. We do not even have the overhead projector … So at the end of the day you still use the old method of teaching.’ (Participant 4, female, nurse educator)
Clinical environment	‘I will rather go to a sister who I can see does things correctly and according to the book … because I will ask her, “Sister I am struggling with this, help me please”. I will trust her to help me do the right thing.’ (Focus group 14, female, student)
	‘Students are really negative towards the clinical area and persons in practice. Here and there you will find a person who stands out where they will say, “This Mr or that sister was really helpful and made an effort to assist us”.’ (Participant 4, female, nurse educator)
Learning opportunities	‘I’ll say in some wards you learn a lot – say, maybe you are placed there for a week or 5 days. And then you learn a lot, but then in another ward, you are placed for 4–5 weeks … but you will go out of that ward not knowing a thing, because in some wards … some sisters are not interested to giving information to the students.’ (Participant 3, female, student nurse)
	‘Yes in the demonstration room if I talk about, for example, CPR or whatever, I cannot do a CPR here. So I need to go to the ward to demonstrate the CPR there on dolls. We do not have CPR dolls here. We do not have a simulation room for midwifery setup here – we do not have the resources.’ (Participant 2, female, nurse educator)
Maintain standards	‘That is when they lack trust in us because if you have high standards, they think you are the cruel one. And that one that has low standards is the good one. So at the end of the day we do not produce the product … you know the well behaved, focused students because we are having double standards.’ (Participant 3, female, nurse educator)
	‘… where you find rules and regulations you feel safe. You know what will happen next.’ (Participant 12, female, student nurse)
**Trusting relationships and trust in quality teaching and learning**	
Product trust	‘Trust grows in you. It is very nice to see if a patient was in a ward where you were working, and he was very sick and then after the treatment, it boosts your self-esteem. You grow up, you can put up a drip, you can stitch, and you know, you get more responsibilities; you get more exposed to a lot of things.’ (Participant 3, female, student nurse)
	‘And we are also worried, we are going to join that force, where we experience the problems, so meaning we maybe end up being part of that problems … We will become unpopular professional nurses if you do not go along with the force … you become unpopular.’ (Focus group 10, female, student))
Programme trust	‘Yes, when you call yourself a professional nurse, you can go overseas and say yes, I have trained in (X), yes it is very high.’ (Participant 3, female, student nurse)
	‘For me personally it is a matter of, you are under supervision, now you realise the fact that next year, somebody is going to be under my supervision. That is the main thing that is worrying me. Next year, somebody is going to say, ‘Sister, what do I do with one, two, and three four five?’ And not like the past 4 years … we will start learning from next year …’ (Focus group 6, female, student)

Concept synthesis (Walker & Avant 1995) was utilised to develop the model. Steps involved refining the model based on clarity, simplicity and consistency; appropriateness and relevance; comprehensiveness; adaptability and generalisability; practicality and usefulness; accessibility; importance for education, practice and research and transferability. Based on the findings from the data, a literature review and inductive and deductive reasoning during the model development process, the educator, student and professional nurses featured as the role players in the model. The model comprises five components, namely nursing education context, professional relations ,expectations of the role players in nursing education, creating a conducive teaching and learning environment and trusting relationships and trust in quality teaching and learning.

### Nursing education environment

**FIGURE 1:**
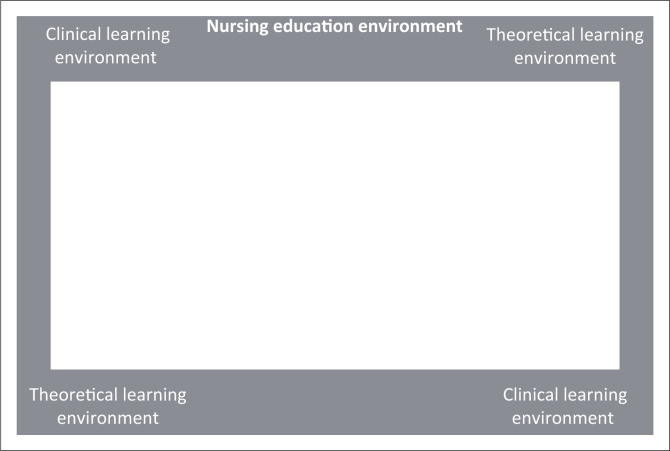
Nursing education environment.

The rectangle in Figure 1 refers to a stable and controlled nursing education environment with conformity and calmness where teaching and learning take place. The role players and all nursing education activities are directed to comply with the minimum requirements of knowledge and skills for a professional nurse. The colour grey in Figure 1 reflects stability and calmness that forms the basis from where professional relations develop.

### Professional relations

**FIGURE 2:**
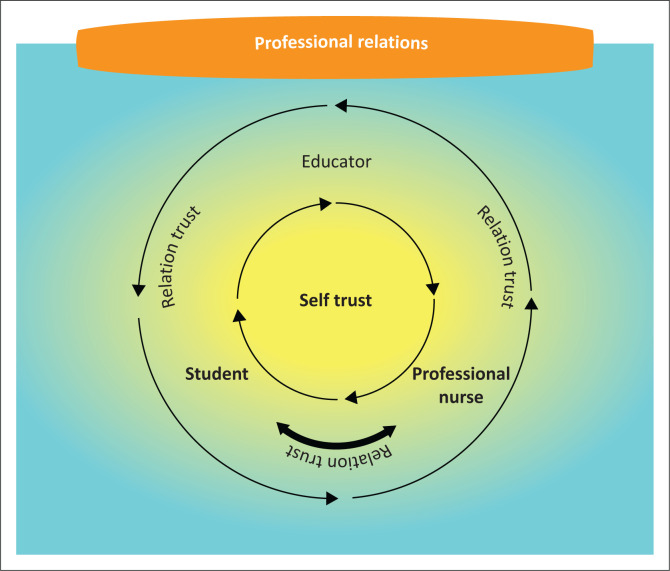
Professional relations.

The second component that relates to confidence and balance are situated in the centre of the model and indicated with a light orange colour. Figure 2 illustrates that trust originates from self-trust. Self-trust is reflected with a yellow colour that represents the awakening of each role player’s confidence and optimism and serves as the foundation of all trusting relationships. The professional relations component encourages balanced trusting relationships with reciprocal self-respect and respect for others. The colour yellow was chosen as it represents enthusiasm and energy. In Table 1, participants’ responses confirmed that trust develops initially from self-trust, which enables the development of healthy interpersonal relationships amongst role players in the teaching and learning environment. Interpersonal relationships and trust develop through interaction amongst role players.

### Expectations of role players in nursing education

**FIGURE 3:**
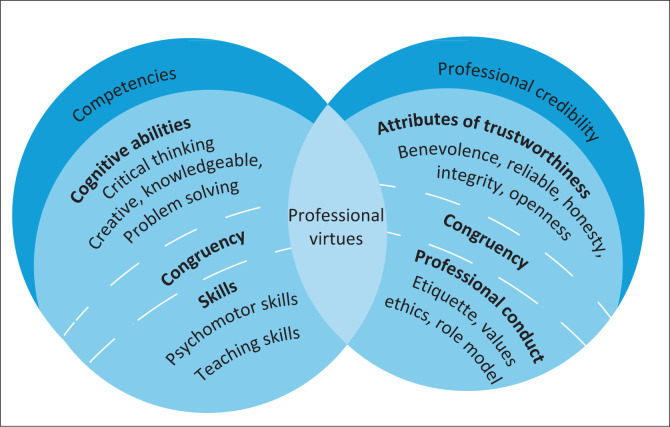
Expectations of the role players in nursing education.

Table 1 reflects core verbal quotes that participants verbalised regarding the expectations of role players in teaching and learning. These expectations include categories of competencies, professional credibility, professional virtues and congruency which support the third component. In Figure 3, the colour blue demonstrates the expectations of role players in nursing education, which represents faith, truth, loyalty, integrity and intelligence. Blue symbolises responsibility, reliability, care and trust.

The competencies of role players establish trust. Cognitive abilities refer to cognitive processes such as knowledge, expert knowledge and creativity that foster mutual trust amongst role players. All role players should have skills such as interpersonal skills, psychomotor skills and sufficient experiences to integrate theory and practice. From competencies, the appropriate conduct of role players supports trusting relationships.

Trust in role players is established through **professional credibility**. Attributes of trustworthiness entail benevolence with caring, fairness and support. Integrity and honesty are signs of reliability that enhance trust. Openness is the sharing of thoughts and feelings that creates positive trusting experiences. Professional conduct is achieved through visible role modelling behaviour such as etiquette, values and adherence to ethical codes.

**Professional virtues,** as illustrated in Figure 3, share properties of cognitive abilities, skills, attributes of trustworthiness, as well as professional and ethical behaviour of the role players. Trust amongst role players is promoted through constant professional virtues.

**Congruency** is achieved with predictability and consistency in the behaviour and skills of role players. Predictions of behaviour, emotions, actions and fairness is based on the consistency demonstrated by role players. Figure 3 illustrates congruency with a half circle that unites all the categories of expectations of role players in the nursing education context.

### Creating a conducive teaching and learning environment

**FIGURE 4:**
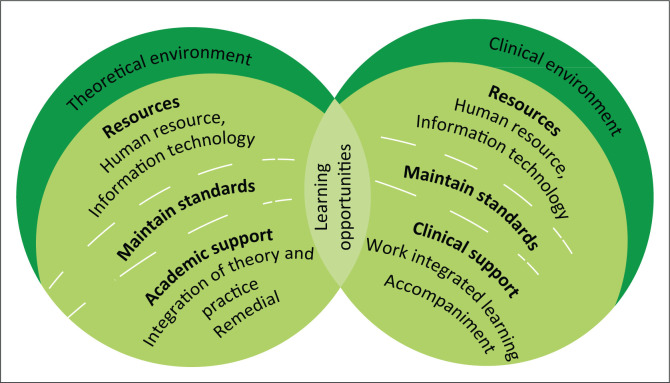
Creating a conducive teaching and learning environment.

Growth, stability, harmony, perseverance and compassion and nurturing are symbolised in the colour green. In Table 1, verbal responses from participants represent the views of role players on how to create a conducive teaching and learning environment. Figure 4 shows the fourth component with the theoretical environment, clinical environment, learning opportunities and standards categories.

Trust in the **theoretical environment** requires adequate human and equipment resources. Qualified educators and available resources such as equipped libraries and sufficient technology promote trust in the theoretical environment. Educators who provide academic support with expected competencies and standards promote trusting relationships. Adequate equipment in classrooms and simulations rooms foster trust in learning opportunities.

Human resources and equipment are the resources in the **clinical environment**. Enough qualified educators and professional nurses for education and training are required in the clinical learning environment. Sufficient medical stock and equipment are needed to create an environment conducive to learning. Clinical support focuses on the accompaniment students receive that ensures clinical learning opportunities.

**Learning opportunities** share properties of the theoretical and clinical environment. Theoretical learning opportunities are obtained in classrooms and simulation rooms and clinical areas with standardised technology and equipment. Trust in learning opportunities is obtained when theory and practice are integrated.

**Standards** are needed for trust in teaching and learning. The knowledge and skills of the role players influences the standards. Availability of information technology, libraries, resource centres, equipment and procedures support expected standards. In the clinical environment, standards are influenced by available equipment and stock to render quality nursing care. Maintaining standards provide the consistency that fosters trust in quality teaching and learning.

### Trusting relationships and trust in quality teaching and learning

**FIGURE 5:**
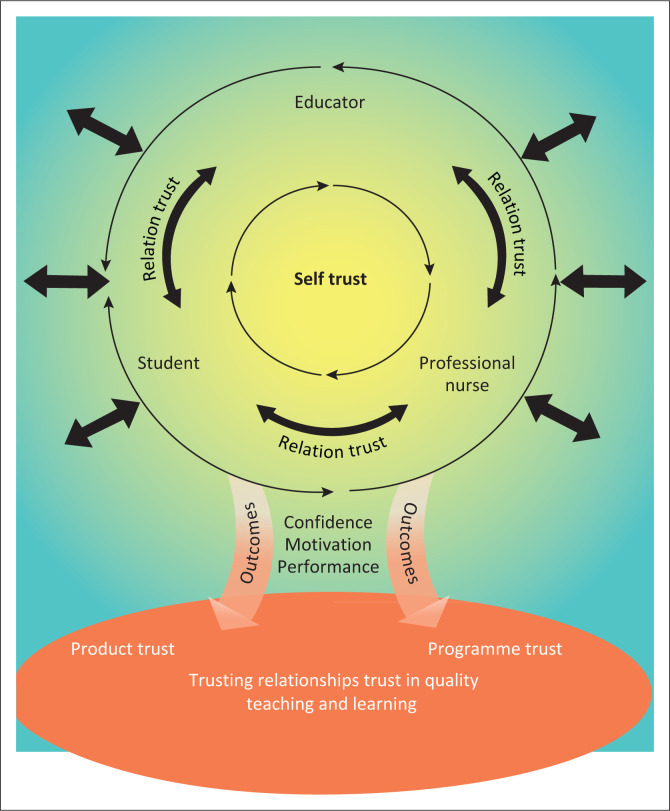
Trusting relationships and trust in quality teaching and learning.

The final component, as represented in Figure 5 in the bottom section (oval), refers to meaning, willpower, leadership and confidence. The verbal responses of participants in Table 1 support the importance of the outcome of trust, namely the trust in the professional nurse as the outcome, product and trust in the programme. The figure illustrates that trust originates centrally from self-trust of the role players. Based on the interaction amongst them, relationship trust develops. Central and at the core of the trust and trusting relationships are willpower, confidence, motivation, passion and better performance. Professional relations amongst role players, relationship trust and trust in the newly registered nurse are provided through compliances of the expectations in role players.

### Synthesis of the components

**FIGURE 6:**
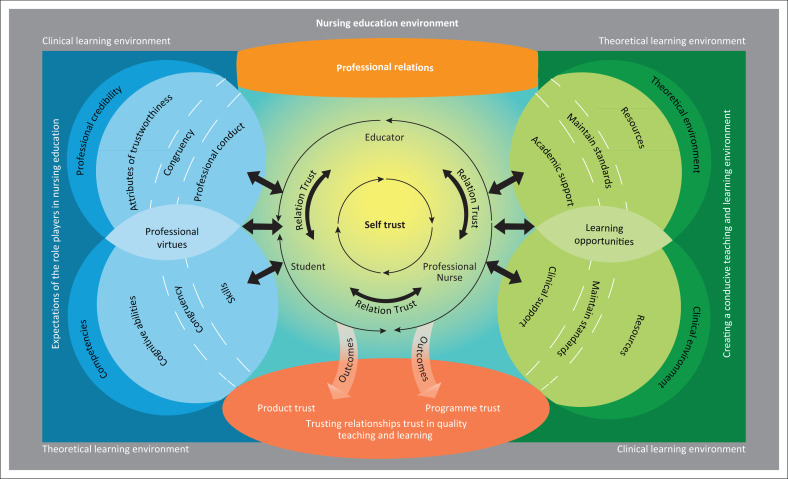
A model for trust in the nursing education environment.

Figure 6 illustrates how the model was created after the synthesis of the components. In the first components the nursing teaching and learning activities take place in the theoretical and clinical environment. The second component, in the centre with professional relations, symbolises self-trust emanating from the centre and trusting relationships developed through the interaction amongst role players. The third component, expectations of role players are fitted on the left. ‘Professional relationships’ and ‘expectations of role players’ are linked with each other and the relationship between the two components are illustrated. The component ‘creating a conducive teaching and learning environment’ is placed on the right side of the model. Component ‘creating an environment conducive to teaching and learning’ links to the ‘professional relations’ component. Each role player is involved in the theoretical and clinical environment for providing and ensuring effective learning opportunities on required standards. Finally, in the middle and at the bottom of model is the ‘professional relations’ component. The model illustrated an equilibrium of the components. On the lateral sides are: expectations of role players and creating a conducive teaching and learning environment. The equilibrium promotes confidence, motivation and performances of the role players. On the left side, the component ‘trusting relationships and trust in quality teaching and learning’, links with the expectations of the role players that indicates that if the role players meet the expected competencies and professional credibility, there will be trust in the newly registered nurse. On the right side, the final component links to the environment conducive to teaching and learning, which ensures the outcome of programme trust. Hence, the model for trust and trusting relationship illustrates an equilibrium with the final outcome as trusting relationships and trust in quality teaching and learning.

## Discussion

The substantive model developed in this study portrays the following criteria: systematic relationships between concepts and links between categories, variations to hold true to different conditions and circumstances, comprehensiveness through psychological and/or social processes and significance that will withstand time. The implementation of the model serves as a guide and provides a structure to improve trust in the nursing education context. Trust and trusting relationships are built by enhancing the climate in nursing education, ensuring sound teaching and learning practices, and ensuring a ‘safe’ environment.

Trust and trusting relationships are formed through various processes that require self-trust and relation trust. Self-trust is essential for relation trust to develop. Findings illustrated that confidence is established in role players through self-trust. Trust is the foundation and core element of an individual’s perception and the internal balance of the person is related to self-trust (Pokrovskaia, Snisarenko & Golohvastov 2016). Role players believing in themselves, having self-discipline and self-trust, are crucial during theoretical and clinical education activities to establish relation trust. Covey and Link (2012) emphasised that an individual acquires credibility by means of beliefs that he or she is competent with a good intent and character. For nursing education, self-trust must be sufficient and the role players’ personality and psychological stability establish confidence in the development of interpersonal relationships. Nursing education institutions should have trusting atmospheres to stabilise interpersonal trust. The foundation for developing trusting relationships emanated from reciprocal interactions amongst role players. Ng (2015) construed that quality education is linked to quality relationships. Evidence in this study reveals that supportive and caring relationships ensure quality of nursing education. A positive atmosphere during teaching and learning depends on the trust. The model provides information on how a positive atmosphere is created through relational processes.

Observable competencies and ethical behaviour of role players foster professional trust and professionalism. Alexopoulos and Buckley (2013) stated that trust is based on shared experiences, skills and competencies. The findings highlighted that educators and professional nurses should have sufficient theoretical knowledge of theory and clinical experience to integrate theory and practice with creative teaching methods. The findings also revealed that students’ trusting experiences enhance enthusiasm, active participation, creativity and self-discipline for improved performances. Furthermore, profound personal value systems, good characters, professional values and moral authority inspire trust. The value systems form part of the model and also indicate the relationship between and amongst the different systems. Choudhury and Barooah (2016) mentioned that character strengths such as teamwork, excellence, curiosity, creativity, gratitude, fairness, hope, honesty, leadership, kindness, self-regulation and love of learning are essential expectations. The foundation of professional trust is build on attributes of trustworthiness such as openness, honesty, reliability and benevolence that will reflect professionalism with ethical values and actions. This is supported by McLemore (2014), who stated that trust is refined in a chain, from values to motives, then following from motives to action. Expected professional conduct requires internalised personal and professional values, merits and ethical codes. These traits steer expected professional behaviour in trusting relationships. The latter will result in role players’ credibility to obtain professional trust. Professional credibility is essential for trust and needs to reflect consistency. Tschannen-Moran (2014) remarked that reliability arises when consistency is clear. The model displays the consistency as an important aspect of trustworthiness and congruency of actions, intentions and emotions as proof of self-control and trustworthiness in a conducive teaching and learning environment.

Learning opportunities are key to the theoretical and clinical environments, as depicted in the model. The educator as an important role player is expected to offer adequate learning opportunities in the teaching and learning environment. Quality teaching and learning requires creative and innovative teaching strategies. Ng (2015) showed that quality education is obtainable by implementing effective teaching and learning procedures. Bossons, Kourdi and Sartain (2012) specified creative, innovative ideas to create critical thinking and problem-solving opportunities with students. Trust is fostered through creativity to promote learning through the linking of theory and practice. Kelly, Berragan and Husebø (2016) concluded that effective learning experiences can be obtained through creative simulations and improve adapting to the real clinical learning environment. Support, caring attitudes, availability of educators in the theoretical environment and accompaniment in clinical areas support and motivate students for active participation to obtain the necessary learning opportunities. The model shows the relationship between creating a conducive teaching and learning environment, the learning opportunities, and the development of self-trust and relation trust.

Technology, proper infrastructure and resources in the teaching and learning environment support the necessary teaching strategies and required learning opportunities. Burkett (2016) indicated that a teaching environment with proper infrastructure and functional technology creates an exciting environment for excellence in learning and nurtures strong relationships between students and educators. With proper teaching strategies and the availability of sufficient resources during education and training, standards will be secured in the nursing education environment. Hurley’s Decision to Trust Model (Hurley 2012) emphasised that trust is ensured when similarities occur in a specific context. Therefore, in a conducive teaching and learning environment where expected outcomes have to be attained, uniformity of standards is a requirement. The model shows the overarching importance of a conducive environment together with the expectations of the role players in nursing education in both the theoretical and clinical learning environment.

The role of professional virtues in ensuring trust is displayed in the model as integrated into professional credibility and competencies. These virtues include attributes of trustworthiness, congruency, professional conduct, skills and cognitive abilities. The outcomes of trust create open relationships and communication where role players build their own self-confidence, self-trust, self-regard, motivation and gratification and competence. Uhrenfeldt and Hall (2015) believed that trust is a key factor for job satisfaction, whilst Dinç and Gastmans (2013) believed that trust is the positive outcome regarding professional roles. Holland (2015) believed that relation trust in a teaching and learning environment may increase better achievements. As a result of the latter’s conclusions and based on the study’s findings, the model includes an illustration of what the outcome of self-trust and relation trust is. Programme and product trust flows from the trusting relations. The outcome of trust in nursing education relies on the inner self-trust, relation trust, positive experiences from the professionalism of role players, and an environment with sufficient standards that ensure excellence in teaching and learning.

The graphic presentation of the model shows the relationships amongst and between concepts, processes, attributes, role players, environment and outcomes. Overlapping components in the graphic illustration show the importance of interrelated processes and relationships, with an overall aim of quality in teaching and learning.

Some limitations were identified. Only 4-year nursing programme students in their third- and fourth-year levels of study were included. The first- and second-year students were excluded and their views might have been different because of limited experiences in nursing education. In this study, professional nurses in the clinical environment were excluded from the population.

Recommendations for NEIs include that developing and maintaining trust and trusting relationships amongst the role players in nursing education context are key to quality teaching and learning. The mission and vision of an institution should highlight trust as a key factor in education and training. Role players need support in developing self-trust and extend relation trust to each other that will inspire motivation, self-confidence and improve performances. Extending self-trust to trusting relationships with role players through visible expected competencies and professionalism are important factors in creating a conducive environment. Verification of these expectations enhances trust in role players and promotes standards of academic and clinical learning opportunities. Sufficient resources to integrate theory and practice during work-integrated learning enhance the experiences during learning opportunities. Conducive teaching and learning environments could be developed based on this model to foster trust in the programme.

## Conclusion

Nursing is perceived as the backbone of the society and an essential career. Therefore, it is vital to have trust in the teaching and learning environment and the education and training of professional nurses. Essential aspects of nursing education and related processes are illustrated comprehensively in the model. Important expectations of role players that contribute to building reciprocal trust are demonstrated. Implementation of this model in the theoretical environment and clinical facilities will emphasise standards and adequate resources that promote an environment conducive to students’ learning opportunities. The fostering of trust and trusting relationships amongst the stakeholders of nursing education will increase a trusting culture in the nursing context with an overall aim of quality teaching and learning.
